# Arthroscopic Management of Shoulder Osteoarthritis

**DOI:** 10.2174/1874325000802010023

**Published:** 2008-02-21

**Authors:** Michael S George

**Affiliations:** KSF Orthopaedic Center, 17270 Red Oak Drive, Houston, TX 77090, USA

## Abstract

Osteoarthritis (OA) can cause severe pain and dysfunction of the shoulder. When conservative treatment fails and operative treatments such as shoulder arthroplasty and open glenohumeral resurfacing are not advisable, shoulder arthroscopy may be used to treat shoulder OA. Arthroscopic treatment of concomitant pathology in the shoulder including subacromial decompression, labral repair, capsular release, microfracture, and distal clavicle excision have been shown to yield good results when combined with glenohumeral debridement in the treatment of shoulder OA. Arthroscopic glenohumeral resurfacing has recently been described and has shown encouraging results. Arthroscopic treatment appears to have better results in shoulders with a lesser degree of osteoarthritis.

## INTRODUCTION

Osteoarthritis (OA) of the shoulder affects an estimated 20% of the elderly population [1]. The management of shoulder arthritis in the elderly is well established. Initial nonoperative measures include anti-inflammatory medications, exercise, physical therapy, and injections. When conservative treatment fails, severe OA can be well treated with shoulder arthroplasty. Approximately 14000 shoulder arthroplasties are performed annually for treatment of OA in America [2]. Long term results of shoulder arthroplasty in the treatment of OA in the elderly are encouraging [3].

Shoulder arthroplasty in younger patients has not been as successful as it has been in the elderly. Active patients with heavy lifting requirements place more stress on the shoulder arthroplasty causing earlier implant failure. Complications such as implant loosening, dislocation, fracture, and persistent pain are more common in younger patients. Sperling reported on sixty-two hemiarthroplasties and twenty-nine total shoulder arthroplasties performed in patients under fifty years old with a minimum 15-year follow-up. Only 44% of these patients had excellent or satisfactory results [4].

Given the poor results of shoulder arthroplasty in young patients, alternative surgical treatments may be a viable option. Other factors that may influence patients to delay shoulder arthroplasty despite severe OA include heavy lifting requirements, active labor, or desire to avoid major surgery. Surgical alternatives to shoulder arthroplasty in the treatment of shoulder OA include tendon transfers, glenoid resurfacing, and shoulder arthroscopy. Shoulder arthroscopy is a useful adjunct to the treatment of shoulder OA in patients for whom shoulder arthroplasty may be inappropriate.

## PATHOPHYSIOLOGY

Shoulder OA may occur as a primary or secondary process. Primary OA is less common than OA of the knee or hip. The onset of pain is usually gradual, although patients may report an inciting injury that exacerbates the symptoms. Secondary OA occurs as the result of trauma or deformation of the glenohumeral joint. Pathological changes of the arthritic glenohumeral joint include labral degeneration, loose bodies, articular cartilage defects, and osteophytes. Coexisting pathology may also include adhesive capsulitis and subacromial bursitis [5].

Shoulder instability is an important cause of secondary OA in young people. Buscayret, *et al.* reported on 570 patients who underwent a shoulder stabilization procedure. The preoperative incidence of glenohumeral arthritis was 9%. Of patients with no preoperative arthritis, an additional 20% developed postoperative arthritis. Risk factors for the development of arthritis in this group included older age at the initial dislocation and at surgery, increased number of dislocations, osseous glenoid rim lesions, and Hill-Sachs lesions [6]. Cameron, *et al.* reported on 422 patients who underwent arthroscopic shoulder stabilization. Older age and longer time from injury to surgery were the greatest predictors of preoperative OA. There was no association between the direction of instability and the presence of OA [7].

The relationship between Hill-Sachs deformity and the development of OA is unclear [6, 8]. Larger lesions with more osteochondral damage appear more likely to progress to more diffuse degenerative changes, although it is not clear if there is a critical size of defect that leads to more rapid OA. Osteochondritis dissecans is another rarely reported cause of articular cartilage defects in the glenohumeral joint [9, 10], although it appears to be much less common than in the knee.

Recently, iatrogenic chondrolysis has been reported in shoulders treated with thermal capsulorrhaphy [11]. Thermal injury to chondrocyte cells may result in cellular death, chondrolysis, and advanced degenerative changes. The combination of thermal damage and shoulder instability may accelerate the process of chondral degeneration and arthritic changes.

## DIAGNOSIS OF SHOULDER OSTEOARTHRITIS

The diagnosis of shoulder OA is based on patient history, physical examination, and radiographic imaging. Patients with shoulder OA typically complain of chronic pain with an insidious onset. Shoulder stiffness, pain in the morning and with weather changes, and pain with increased activity are common complaints. Patients may complain of a specific injury that exacerbates the pain and stiffness.

Physical examination of the shoulder starts with a thorough inspection of the entire shoulder girdle. Scars from previous trauma or surgery are visualized. The supraspinatus and infraspinatus are inspected for atrophy that could indicate rotator cuff pathology. The shoulder is checked for posture and for scapular winging which could affect the biomechanics of the shoulder. Deformity consistent with large osteophytes or previous trauma is determined and compared to the contralateral side.

Shoulder OA is frequently complicated by secondary adhesive capsulitis due to incongruous joint surfaces, osteophytes, and capsular scarring. Range of motion is assessed both passively and actively. Passive abduction and forward flexion are tested. Internal and external rotation is tested in adduction and 90 degrees of abduction. Palpable or audible clicks with shoulder motion may indicate bursitis, biceps tendon pathology, or osteophytes.

The rotator cuff is examined to determine the contribution of subacromial bursitis to the patient’s symptoms. Rotator cuff strength evaluation is performed by testing active external rotation, internal rotation, abduction, and forward flexion strength. Subacromial impingement syndrome is tested using the Hawkins and Neer impingement tests. The long head of the biceps tendon is examined with the Speed and Yergason tests. In cases of shoulder instability, the anterior and posterior apprehension and relocation tests are performed.

Acromioclavicular joint arthritis may also contribute to the pain associated with shoulder arthritis. The acromioclavicular joint is palpated and assessed for swelling, deformity, and instability. The cross-body adduction test is positive when pain is elicited in the acromioclavicular joint.

Ellman described the “compression-rotation test” for examination of the arthritic shoulder. The patient is placed in the lateral decubitus position with the unaffected side down. The humeral head is compressed into the glenoid and the shoulder is internally and externally rotated. Pain is elicited as the arthritic glenohumeral joint surfaces are compressed together [12]. The test may be more specific after a subacromial lidocaine injection [8] to lessen the contribution of subacromial bursitis to a positive test.

Plain radiographs should include true glenohumeral AP, scapular “Y”, and axillary x-rays [8]. Weinstein, *et al.* described a radiographic classification of shoulder OA. Stage I is normal radiographs, but with arthroscopic evidence of articular cartilage changes. Stage II is minimal joint space narrowing with concentricity of the humeral head and the glenoid. Stage III is moderate joint space narrowing with early inferior osteophyte formation. Stage IV is severe loss of joint space with osteophyte formation and loss of concentricity between humeral head and glenoid [5].

CT scan is a useful radiographic tool to determine the extent of arthritic deformity, osteophyte formation, and glenoid version. MRI may be peformed to evaluate soft tissue structures in the shoulder such as the rotator cuff, biceps tendon, and glenoid labrum, which may contribute to the patient’s symptoms. MRI, however, has been shown to have a high sensitivity but low specificity for determination of articular cartilage lesions in the shoulder [13].

**Fig. (1):**
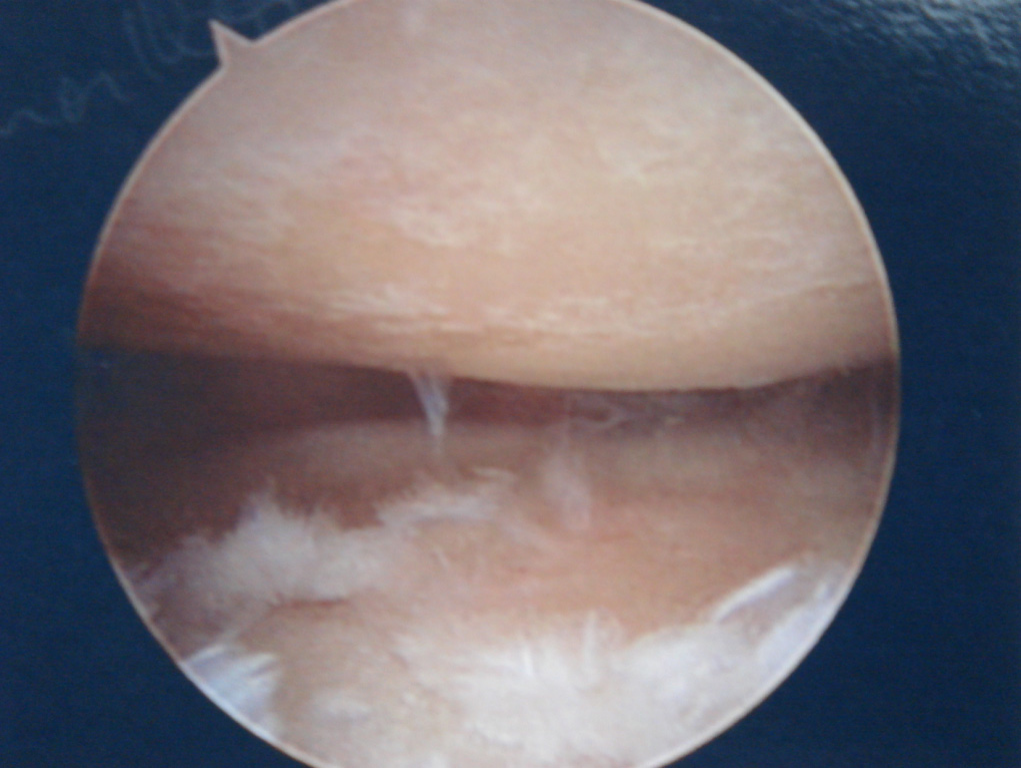
Arthroscopic view of Grade IV chondral changes of the glenoid and humeral head.

On arthroscopy, articular cartilage lesions are typically classified according to the Outerbridge classification, as has been described in the knee. Grade I is softening or blistering of the articular cartilage. Grade II is fissuring and fibrillation of the articular surface. Grade III is deep ulceration of articular cartilage without exposed bone. Grade IV is full thickness cartilage loss with exposed subchondral bone [14] (Fig. **1**).

## INDICATIONS FOR SHOULDER ARTHROSCOPY

Initial treatment of shoulder OA should focus on nonoperative treatments including anti-inflammatory medications, physical therapy, and steroid injections. Early studies regarding viscosupplementation injections are encouraging [15]. Surgical intervention is indicated when conservative treatment fails.

Shoulder arthroscopy is indicated for patients with severe shoulder OA who are not good candidates for shoulder arthroplasty due to young age, activity level, or desire to avoid major surgery. Shoulder arthroscopy avoids long hospitalization, allows a relatively quick recovery, and spares bone and soft tissue, allowing for subsequent arthroplasty if necessary [16, 17].

## ARTHROSCOPIC PROCEDURES

Arthroscopic debridement of the arthritic glenohumeral joint includes removal of loose bodies, chondral flaps, and degenerative tissue. A stable transition zone between degenerative and intact articular cartilage is created using a combination of arthroscopic shavers, baskets, and curettes [8]. All loose articular cartilage is removed, while being careful to leave healthy cartilage intact. Small osteophytes may be removed, however it is not recommended to debride large inferior osteophytes due to the risk of neurovascular injury [18]. It has been hypothesized that arthroscopic lavage and removal of debris may improve pain simply by diluting degenerative enzymes [5]. There are no outcome studies in the literature regarding the outcome of isolated glenohumeral debridement for treatment of shoulder OA.

Subacromial decompression may be beneficial in the treatment of shoulder OA, as subacromial bursitis is frequently present concurrently with glenohumeral OA. Weinstein, *et al.* reported on 25 patients with radiographic Stage II-III changes treated with arthroscopic subacromial bursectomy, debridement, and loose body removal with an average follow-up of 34 months. 9/25 of the patients had previously undetected osteoarthritis. 80% of patients had good or excellent results. Of the 12 patients with preoperative stiffness, 83% of patients had improved motion postoperatively [5]. Ellman, *et al,* performed subacromial decompression and glenohumeral debridement on 18 patients. They noted good results with short-term follow-up [12]. Guyette, *et al. *performed subacromial decompression on 36 patients with a mean 5 year follow-up. The 26/36 patients with Grade I-III changes had a L’Insalata score of 90 at follow-up whereas 10/36 patients with Grade IV changes had a score of 50 at follow-up [19], indicating that the procedure was more successful in patients with less severe OA.

Shoulder OA and adhesive capsulitis may occur simultaneously and can be clinically difficult to differentiate from one another. As in the treatment of primary adhesive capsulitis, arthroscopic capsular release and/or manipulation under anesthesia may successfully regain motion and relieve pain. Cameron, *et al.* reported on 45 patients with grade IV osteochondral lesions treated with arthroscopic debridement with a minimum follow-up of two years. 36% of patients also had an arthroscopic capsular release. The mean patient satisfaction score (0 = not satisfied; 10 = completely satisfied) improved from 0.67 preoperatively to 6.28 at final follow-up. Osteochondral lesions greater than 2 cm were associated with return of pain and failure of this procedure [20]. There are no reports of the outcome of manipulation under anesthesia in the treatment of OA with secondary adhesive capsulitis.

Biologic resurfacing has recently been described for the treatment of glenohumeral arthritis. The goal of this treatment is to interpose a synthetic or biologic scaffold of sufficiently high tensile strength to permit repopulation by host cells [21]. Autogenous fascia lata, anterior shoulder capsule, meniscal allograft, and regenerative tissue matrix have been used successfully in open reconstructive procedures [22, 23]. Arthroscopic resurfacing has recently been described using regenerative tissue matrix. Second look arthroscopy demonstrated fibrocartilage ingrowth at three months postoperatively [21]. Brislin, *et al.* performed arthroscopic resurfacing using a bovine patch in 10 patients with good results overall. Patients averaged an increase of 60 degrees of forward flexion, 50 degrees of abduction [24]. Pennington described an arthroscopic technique of arthroscopic lateral meniscal allograft resurfacing in 10 patients with promising short term results [25].

Isolated osteochondral lesions of the humeral head can be treated with microfracture as is recommended in the knee. In the knee, the ideal lesion for microfracture treatment is an isolated, well contained lesion not exceeding an area of 4 square cm. Loose chondral flaps are removed and a curet is used to remove the calcified cartilage layer. Microfracture awls are used to penetrate the subchondral plate leaving a 3-4 mm osseous bridge between microfracture holes. Bleeding after microfracture leads to a pluripotent mesenchymal clot [26]. Siebold reported on five patients treated with open microfracture and periosteal flap for articular osteochondral defects with a mean 26 month follow-up. At final evaluation, Constant scores improved from 43 preoperatively to 82 postoperatively [27]. Arthroscopic microfracture has also been reported in the treatment of isolated humeral head osteochondral defects with excellent results [9, 10].

Degenerative labral tears are frequently seen at arthroscopy in the arthritic shoulder. Labral flaps and frayed tissue should be debrided. Labral avulsions should be repaired to the glenoid rim, being careful not to imbricate or superiorly shift the capsulolabral complex which can lead to further arthritic changes. The labrum can be carefully repaired in a manner to cover Grade IV lesions on the glenoid rim, possibly slowing their progression [18] (Fig. **2**).

**Fig. (3):**
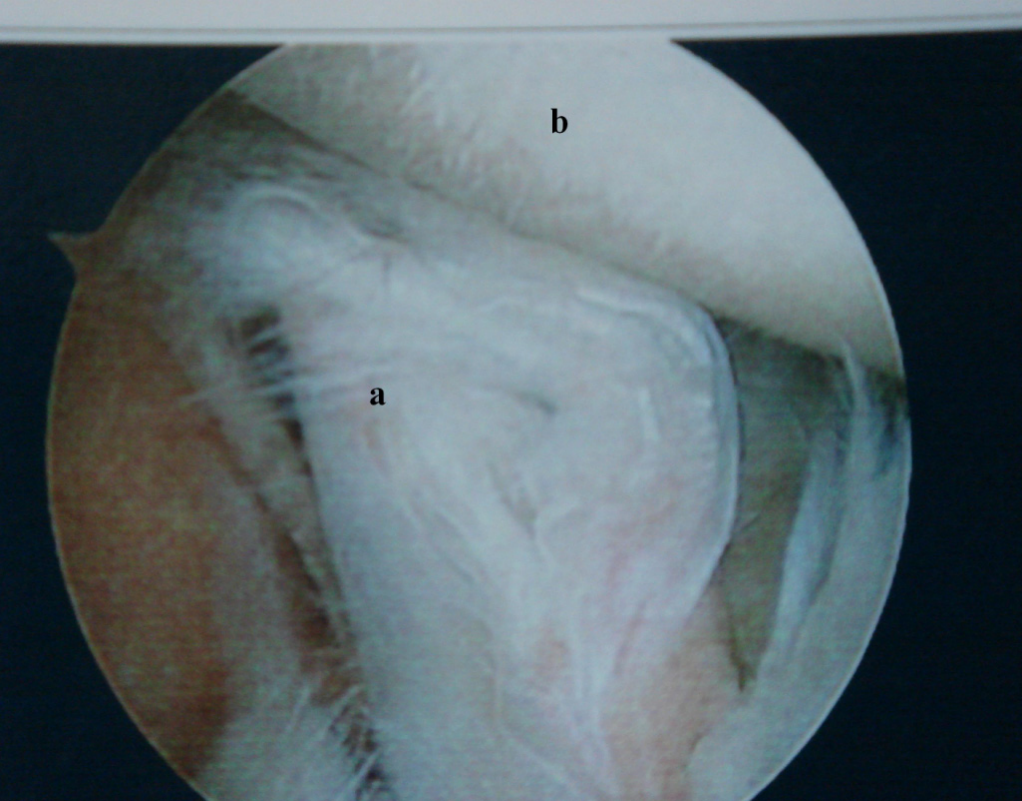
Arthroscopic view of partial thickness tearing of the long head of the biceps tendon (**a**). The humeral head (**b**) has Grade III chondral changes.

Bicipital tenosynovitis may be caused by friction against intraarticular osteophytes. Partial or complete degenerative tearing of the long head of the biceps tendon is also commonly seen in the degenerative shoulder (Fig. **3**). Biceps tenotomy or tenodesis may be necessary when macroscopic tendonopathy is present [28].

Symptomatic acromioclavicular joint degeneration is also commonly seen concurrently with glenohumeral OA and may be successfully treated with arthroscopic distal clavicle excision. The long term results of arthroscopic distal clavicle excision with concomitant subacromial decompression in shoulders without glenohumeral OA are excellent [29]. There are no outcome studies in the literature regarding repair of degenerative labral tears, biceps tenodesis or tenotomy, or distal clavicle excision in the treatment of shoulder OA.

## CONCLUSIONS

Arthroscopic management of shoulder arthritis is a useful treatment in young or active patients for whom it is advisable to delay shoulder arthroplasty. Shoulder OA is frequently seen concurrently with subacromial bursitis, acromioclavicular joint arthritis, labral tears, tendonopathy of the long head of the biceps tendon, and adhesive capsulitis. Arthroscopic treatment of these concurrent disorders should be combined with debridement of the arthritic glenohumeral joint. Glenohumeral debridement and subacromial decompression appear to have good short term results. Other treatments include capsular release, labral repair, and biceps tenodesis or tenotomy. Biologic resurfacing has recently been described with promising short-term results. Arthroscopic management appears to be more successful in shoulders with a lesser degree of osteoarthritis. Further studies are needed to continue to evaluate the overall efficacy of shoulder arthroscopy in the treatment of shoulder OA.

## Figures and Tables

**Fig. (2a):**
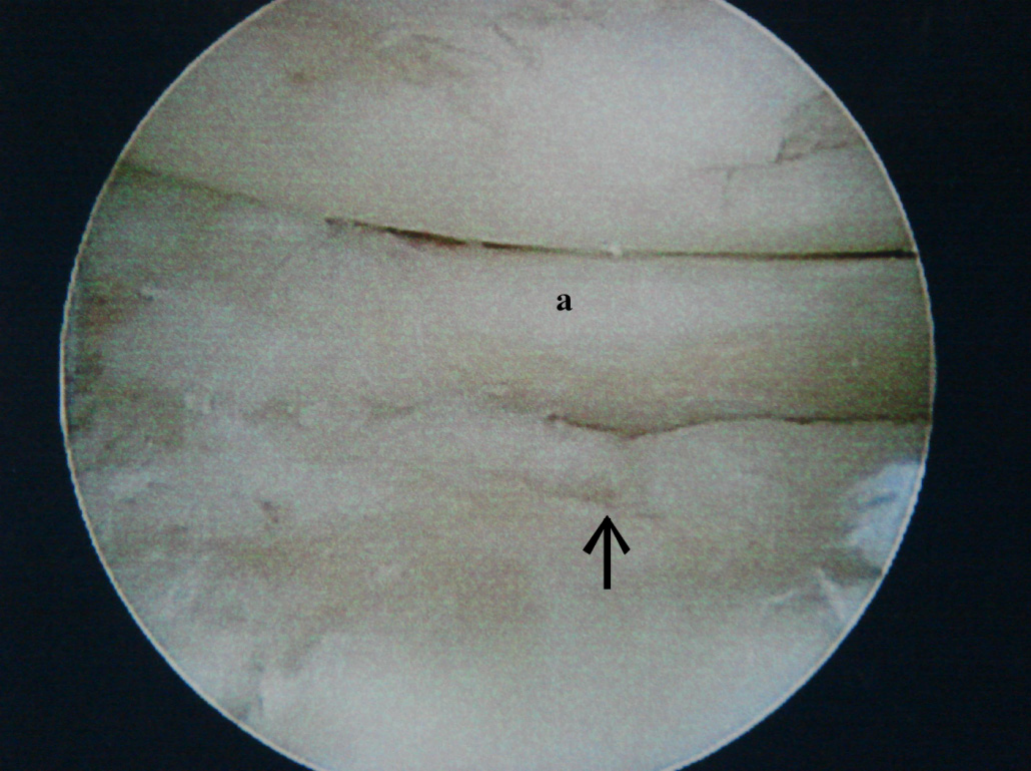
Degenerative anterior labral tear (**a**). The arrow points toward an underlying grade IV chondral defect.

**Fig. (2b):**
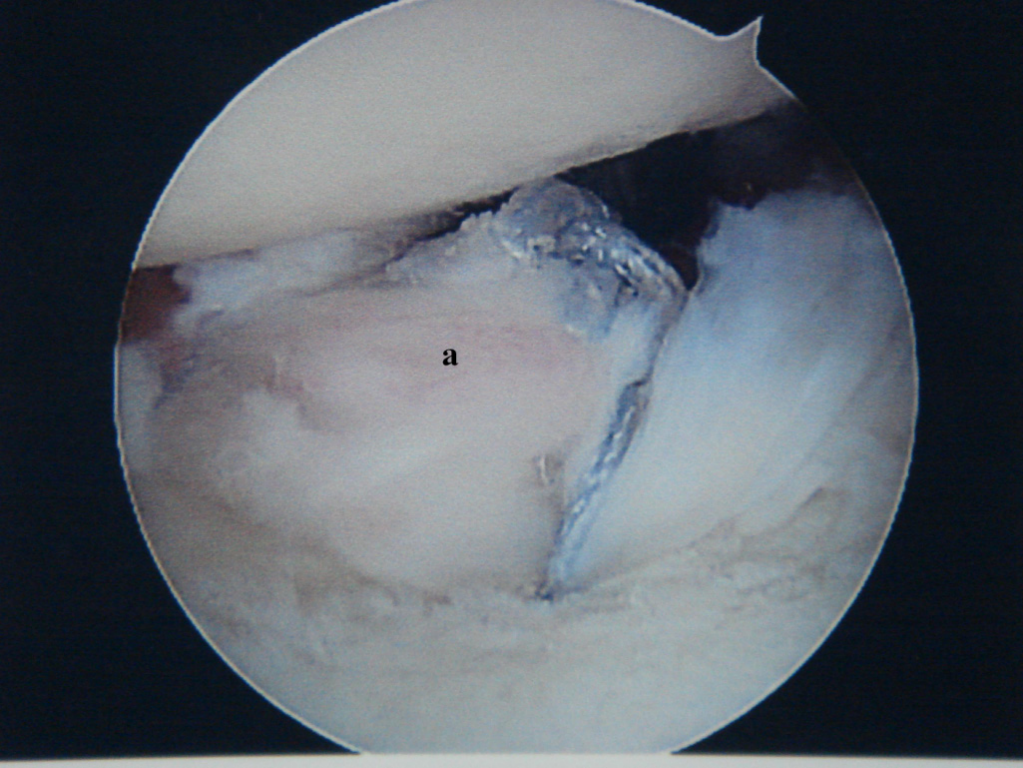
The labrum (**a**) has been repaired to the glenoid rim to cover the grade IV chondral defect.
